# Yield Smarter, Not Harder: Good Practices for Machine Learning of Reaction Outcomes

**DOI:** 10.1021/jacs.6c02213

**Published:** 2026-07-22

**Authors:** Idil Ismail, Gregory A. Landrum, Sereina Riniker

**Affiliations:** Department of Chemistry and Applied Biosciences, 27219 ETH Zurich, Vladimir-Prelog-Weg 2, Zurich 8093, Switzerland

## Abstract

Reaction yield prediction is a longstanding challenge in synthetic chemistry, with broad implications for route planning, scalability, and high-throughput experimentation (HTE). While recent machine learning (ML) approaches have demonstrated promise in modeling reactivity, they often use complex descriptors or deep architectures that are computationally expensive and limit interpretability and scalability. Here, we assess how much information is stored in simpler descriptors and whether model accuracy is improved by increasing the complexity of the descriptors. Using classical ML models trained on descriptors with different complexity levels, we benchmark predictive performance on four publicly available HTE data sets covering three diverse reaction data sets: Buchwald–Hartwig (BH) amination, Suzuki–Miyaura (SM) coupling, and the silicon–amine protocol (SLAP). Our evaluation furthermore discusses (1) generalization via component-wise data splitting, (2) robustness through external validation across data sets, and (3) performance across asymmetric yield distributions characteristic of HTE data. Contrary to conventional expectations, we find that simpler models with interpretable features can achieve competitive performance under rigorous validation protocols. Based on our findings, we formulate good practices for future studies in this area. For example, comparison to low-cost baseline models should become a requirement for future ML studies for reaction-yield prediction.

## Graphical abstract

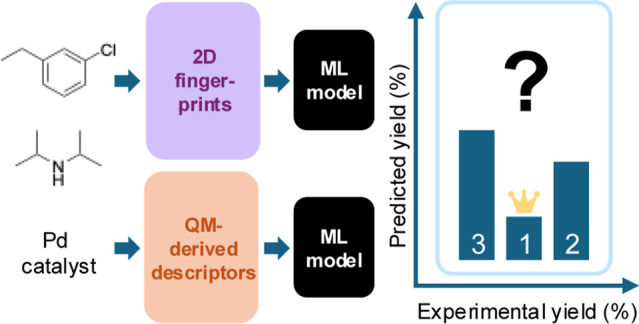

## Introduction

Reaction yield, defined as the amount of product obtained relative to the theoretical maximum based on stoichiometry, is a critical determinant of synthetic efficiency^1^ and directly determines the viability of a synthesis route, especially in multistep or large-scale processes. As such, improving yield remains a core goal in synthetic chemistry, with broad implications for scalability, sustainability, and economic feasibility.^2^ Optimizing reaction conditions to maximize yield typically requires extensive screening across reagents, catalysts, solvents, and temperatures. This process is labor-intensive and resource-demanding and must be repeated for each new substrate or transformation.^3^ As a result, chemists tend to favor well-established transformations, guided by personal experience, literature precedent, and informal heuristics, commonly distilled as personal “cheat-sheets”.^4^ However, these strategies introduce a strong bias toward familiar reactivity and discourage exploration of unconventional or untested routes. Therefore, one of the expectations for computational tools is that they enable broader and at the same time more efficient exploration of reaction space.^5,6^

In recent years, machine learning (ML) for reaction discovery and yield optimization, where models are trained to associate molecular structure and reaction conditions with experimental outcomes, has emerged as an active area of study.^7–21^ The promise of these models is that they can use prior data to reduce the need for exhaustive experimental screening by predicting yields of untested reactions. Many published models were trained on computationally expensive and/or high-dimensional descriptors to encode reaction information, including quantum-chemical features such as orbital energies, steric profiles, and electron density-derived metrics, as well as learned embeddings from graph neural networks or transformer-based architectures.^7,19^ While intellectually attractive (we know that quantum-mechanical (QM) effects determine reactivity), effective within well-defined domains, and able to encode fine-grained reactivity signals, these descriptors incur substantial computational costs. In contrast, simpler representations such as topological fingerprints, 2D physicochemical descriptors, or basic reactant encodings are fast to compute, broadly applicable, and often easier to interpret; however, they are generally assumed to lack sufficient resolution for precise prediction.^21^ This raises a fundamental question: How much descriptor complexity is actually required to build accurate and generalizable ML models for yield prediction? As illustrated in Figure 1, different descriptor classes span a wide range of trade-offs across criteria such as chemical meaningfulness, predictive power, computational efficiency, amount of structural information required, generalizability, and interpretability. No single representation dominates across all axes, and the assumption that more complex descriptors necessarily lead to better performance remains largely untested, particularly under realistic modeling constraints and validation protocols.

**Figure 1:**
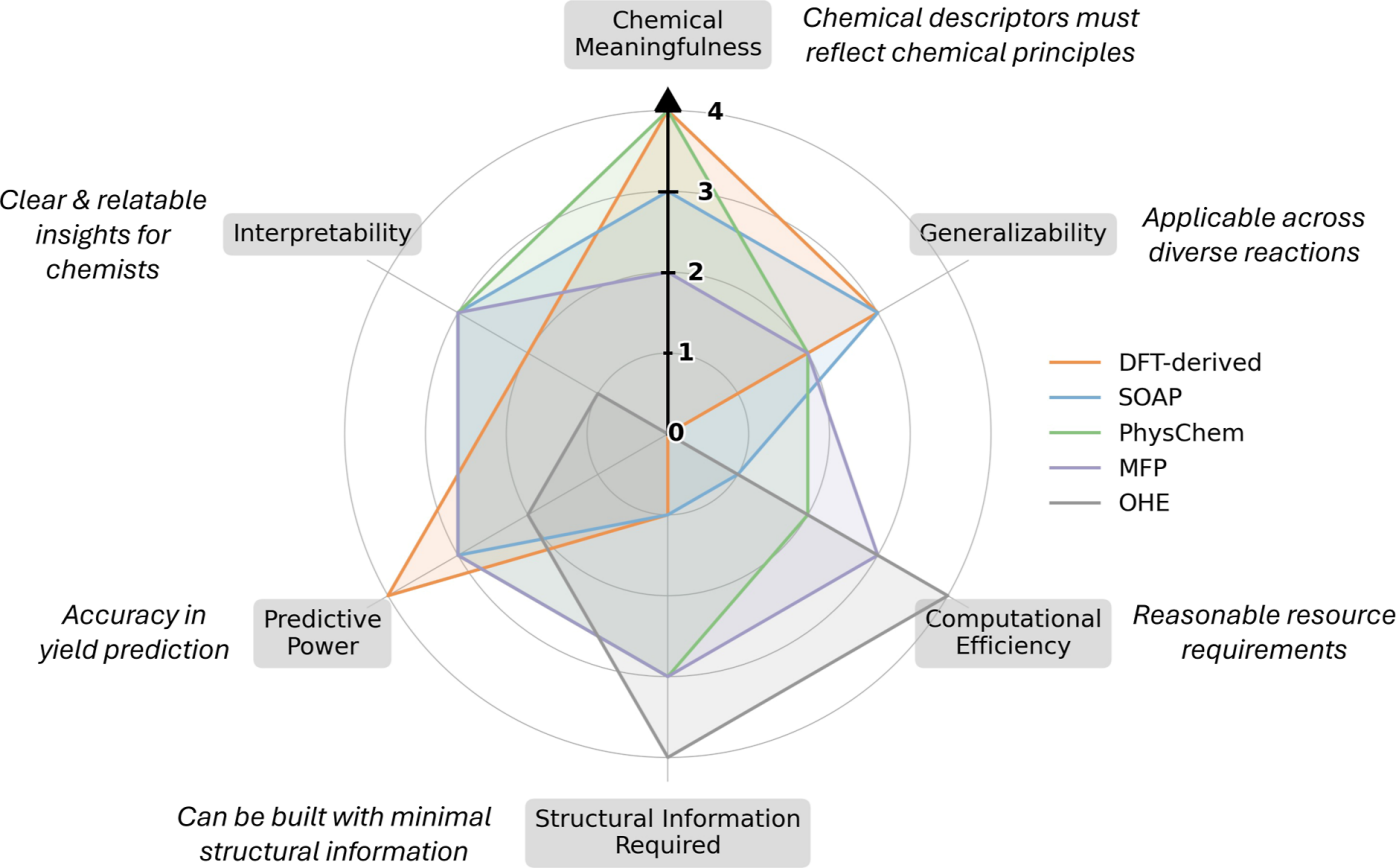
Qualitative comparison of descriptor types evaluated across six criteria: chemical meaningfulness, computational efficiency, amount of structural information required, generalizability, predictive power, and interpretability. Scores are given on a scale from 0 to 4, where higher values are more favorable. The five descriptor types ranked are density functional theory (DFT) derived, smooth overlap of atomic positions (SOAP),^22^ physicochemical properties (PhysChem),^23^ Morgan fingerprints (MFPs),^24^ and one-hot encoding (OHE).^25^

These trade-offs are especially relevant when considering the interpretability and usability of predictive tools in real-world synthetic settings. Chemists are more likely to trust and adopt models that align with chemical intuition and provide rationalizable output. While deep-learning models can encode subtle structural patterns, they often operate as black boxes. In contrast, classical ML models built on low-cost, chemically transparent descriptors can offer greater interpretability and be more easily integrated into existing synthetic planning workflows. As ML becomes increasingly embedded in automated synthesis platforms, simplicity and explainability are not just conveniences but also prerequisites for practical deployment.

Beyond representation, methodological choices in model evaluation also limit the ability to assess the reliability and generalizability of many ML-based yield predictors. A common practice is to use random train–test splits, which means that the test set is drawn from the same distribution as the training set, i.e., structurally identical reactants or products may be part of both training and test sets. Random splitting thus often provides an upper bound on the model performance. It represents the application scenario where a model is used to predict the reaction outcome of a predetermined library of reagents, where the training set is a broadly sampled subset of the possible combinations of reagents. However, if the application scenario of a model is to predict the reaction outcome of reagents not seen during training, this ability to generalize to novel chemistry^26^ needs to be validated. One strategy, discussed in more detail below, for generating such an out-of-distribution test set is to split the data by reaction component identity (e.g., unique aryl halides or nucleophiles).

A further and critical complication arises from the asymmetry of reaction yield distributions. Data sets collected from patents or the literature often exhibit publication bias, containing a (too) high proportion of successful, high-yielding reactions.^27^ In contrast, high-throughput experimentation (HTE) data sets show a distribution shift to lower yields,^7–9^ as illustrated in Figure 2. The higher proportion of reactions with low yields or failed reactions in HTE data sets is not only due to their design intent (i.e., systematic exploration of the combinatorial space of reagents versus a more selective coverage in patent/literature data sets) but also reflects intrinsic microscale experimental variability. As has been demonstrated in high-throughput screening for drug discovery: even small deviations in liquid-handling precision and accuracy can measurably affect assay outcomes, especially for low-potency compounds.^28^ Analogously, in reaction HTE, nanoliter–microliter dispensing errors can introduce significant variance, particularly under marginal conditions.

**Figure 2:**
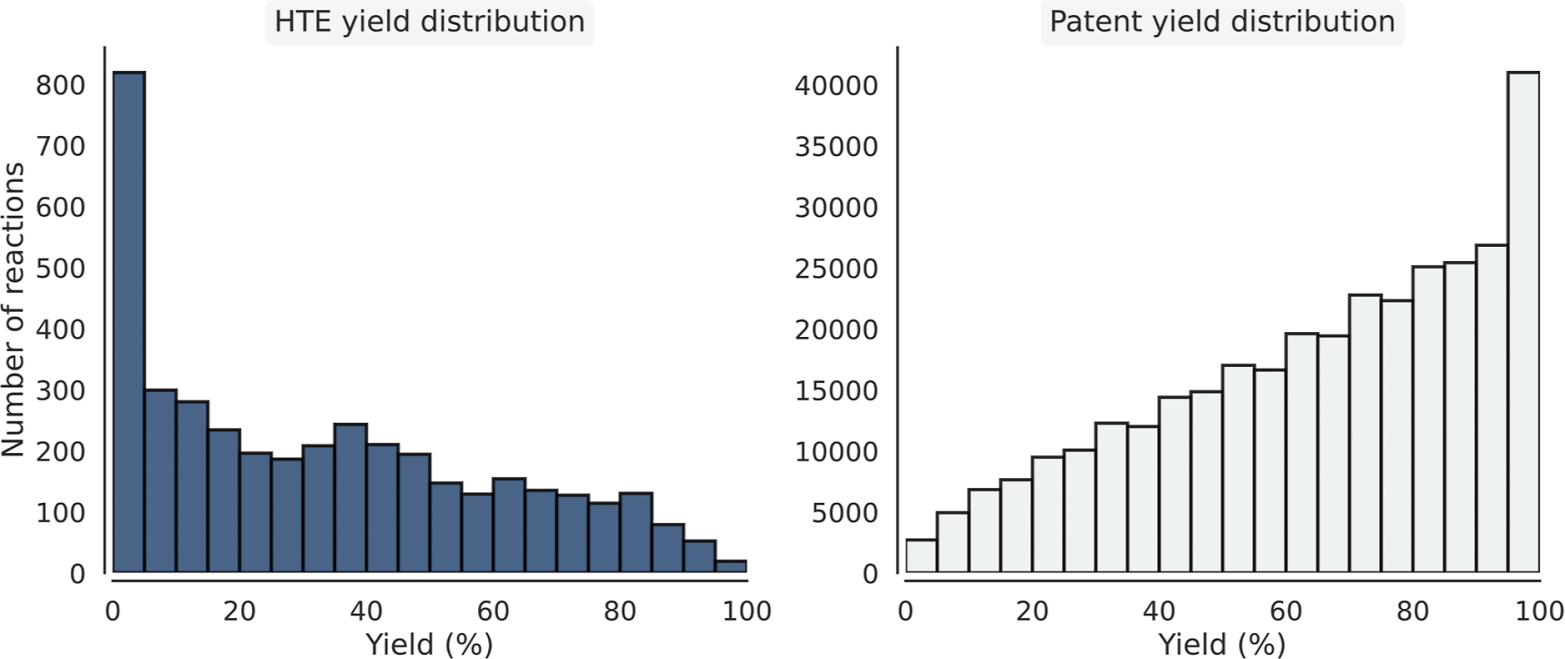
Yield distributions from high-throughput experimentation (HTE, left) and USPTO patents (right). The HTE Buchwald–Hartwig data set of Ahneman et al.^7^ includes a high proportion of low-yield or failed reactions, reflecting the exploratory nature of early-stage development. In contrast, the distribution in the USPTO data set of the same reaction class^27^ is shifted toward high yields, illustrating a publication bias toward successful outcomes.

A final difficulty in modeling reactions is lacking or inconsistent metadata describing important factors like stoichiometry, reaction conditions, reaction times, etc. This is generally not a problem when using an individual HTE data set, where all of the experiments were conducted under the same conditions, but can be quite significant when assembling data sets from patents or the scientific literature.

While HTE data sets pose unique modeling challenges, they also represent the most relevant test cases for real-world applications of yield prediction models. Initiatives such as the Open Reaction Database (ORD) aim to unify successful and unsuccessful reactions and encourage the deposition of well-structured reaction metadata,^29^ thereby reducing the observed biases toward either high-yield or low-yield data points. However, data imbalance is currently a concrete and persistent issue that has been largely overlooked in ML studies of yield prediction.

In this perspective, we examine how much descriptor complexity is necessary to support accurate and generalizable yield prediction and discuss performance evaluation strategies as well as potential approaches to mitigate the problems caused by imbalanced yield distributions. The benchmarking of descriptor complexity is performed using classical ML and four publicly available HTE data sets for three distinct reaction classes: Buchwald–Hartwig amination (BH),^7,8^ Suzuki–Miyaura (SM) coupling,^9^ and the silicon-ligand amine protocol (SLAP).^10^ Based on our analysis, we formulate good practices for future ML studies for yield prediction. In particular, we challenge the prevailing assumption that greater descriptor complexity is generally necessary for predictive success and advocate that comparison to simple, low-cost approaches is a requirement. As the community continues to integrate ML into high-throughput synthesis, automated discovery platforms, and electronic lab notebooks, it is important that we yield smarter, not harder.

### How Much Descriptor Complexity Is Needed?

A key challenge in ML-guided reaction modeling is the choice of molecular representation. Descriptor types vary widely in complexity, computational cost, and information content, and their suitability often depends on the scale and purpose of the modeling task. To evaluate how descriptor complexity influences predictive performance, we benchmarked five distinct molecular representations with increasing complexity on four publicly available HTE data sets using random forest (RF)^25^ regression as the ML algorithm. RFs are fast to train, resistant to overfitting, and perform reliably on small-to medium-sized data sets, making them ideal for benchmarking of reaction prediction models. The performance using other ML algorithms (light gradient boosting machine (LightGBM),^30^ k-nearest neighbors (KNN),^25^ and ridge regression^25^) is provided in Section S4 in the Supporting Information. The HTE data sets cover three different reaction classes: Buchwald–Hartwig amination (data sets BH1 ^7^ and BH2 ^8^), Suzuki–Miyaura coupling (data set SM^9^), and SLAP reactions (data set SL1 ^10^). These HTE data sets were specifically designed to support ML applications, with a particular focus on predicting reaction yields.

Figure 3 illustrates the composition and scope of the four data sets. The BH1 data set from the work of Doyle and co-workers^7^ contains 3955 reactions conducted in the same solvent and using the same amine coupling partner. It remains one of the most comprehensive and widely studied benchmarks for reaction yield prediction. The BH2 data set was published by Denmark and co-workers,^8^ comprising 3359 reactions. Reaction yields were reported for each entry, enabling a fine-grained analysis of how different substrates respond to varying reaction conditions. While the BH1 data set focuses on systematic component variation within a fixed scaffold, the BH2 data set expands the chemical scope by exploring diverse substrate combinations under broader condition settings. The SM data set^9^ comprises 5760 reactions sampled from a combinatorial space of 8624 conditions, generated by varying electrophiles, nucleophiles, palladium catalysts, bases, and solvents. Finally, the SLAP data set SL1 published by Bode and co-workers^10^ contains 1150 reactions, exploring combinations of aldehydes, bifunctional SLAP reagents, and structurally diverse aldehyde partners to enable systematic investigation of three-component coupling. Reactions are defined solely by the identities of the three coupling partners with no variation in reaction conditions. More details on the four HTE data sets can be found in Section S1 in the Supporting Information.

**Figure 3:**
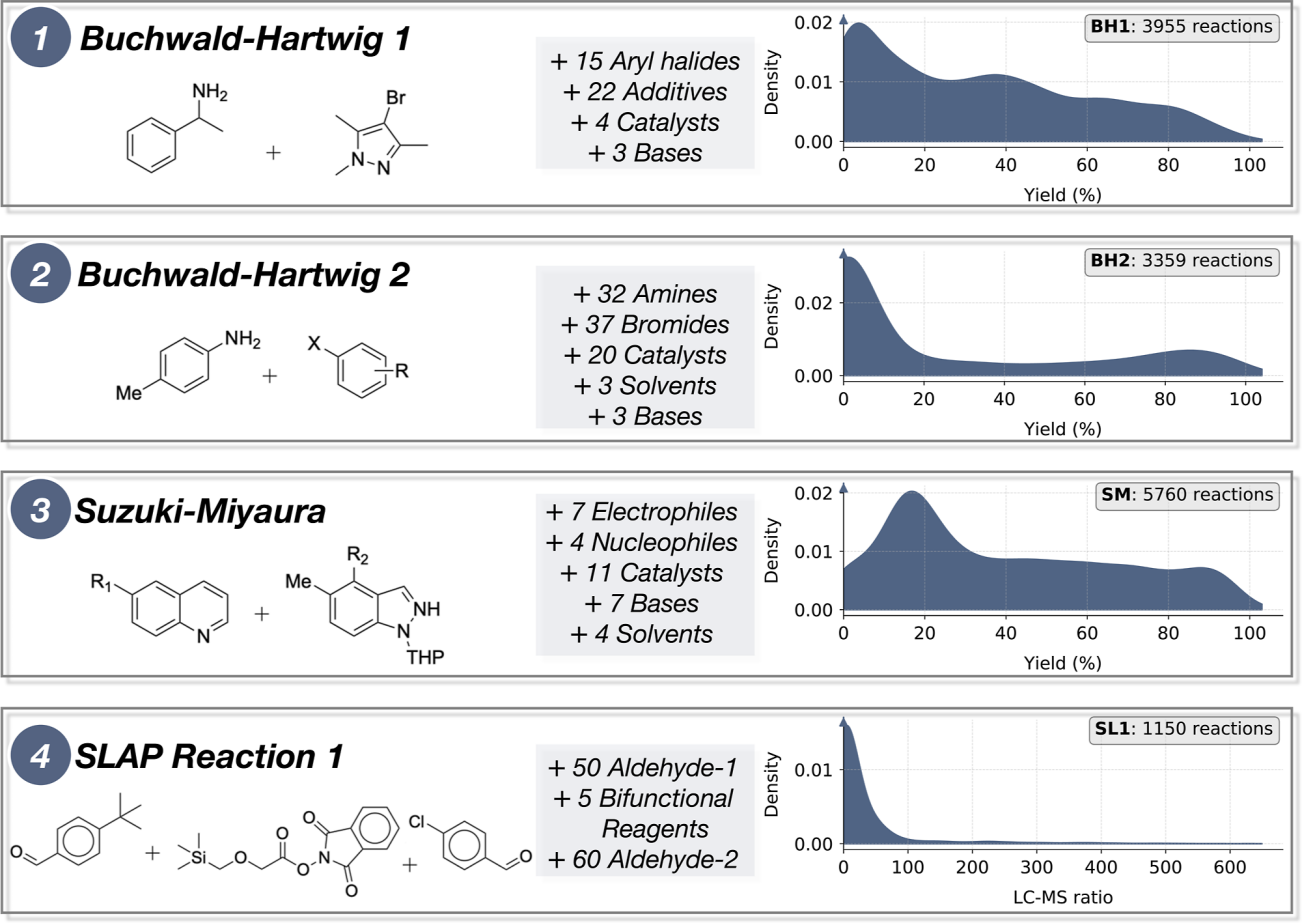
Overview of the four HTE data sets considered in this study, along with statistics on their reaction components: Buchwald–Hartwig amination (BH1 and BH2), Suzuki–Miyaura coupling (SM), and the SLAP reaction (SL1). Kernel density estimates of the observed yield values within each data set are shown on the right.

Our goal was to identify how much improvement in performance is provided by “expensive” descriptors over simpler alternatives and whether simpler descriptors can provide comparable results at a much lower computational cost. The molecular descriptors used in this study, ordered by increasing abstraction from QM-derived to categorical representations, are (Figure 1): (i) density functional theory (DFT)-derived descriptors, (ii) smooth overlap of atomic positions (SOAP),^22^ (iii) 2D physicochemical (PhysChem) descriptors, (iv) Morgan fingerprint count vectors (MFPs),^24^ and (v) one-hot encoding (OHE). DFT-derived descriptors aim to encode a QM representation of a molecule, capturing both electronic and vibrational characteristics. These include frontier orbital energies (*E*_HOMO_, *E*_LUMO_), low-frequency vibrational modes, electrostatic properties from CHELPG charges,^31^ Mulliken charges,^32^ and global reactivity indices such as electronegativity, hardness, and the HOMO–LUMO gap. For the BH1 data set, we used the precomputed DFT-derived descriptors from the original study, while we calculated these descriptors for the other data sets following the same procedure as in ref 7. SOAP descriptors aim to encode the 3D atomic environments within a molecule by projecting local atomic densities onto a basis of radial and spherical harmonics.^22^ This representation is invariant to rotation, translation, and atom indexing, making it well-suited for capturing steric and conformational features. Since the most important 3D structure for any particular species is not known, we chose a single low-energy conformer for each molecule (details of the selection procedure are given in Section S2.2.2 of the Supporting Information). An alternative approach that has been explored in the literature is to employ multiple conformers for each molecule in combination with multiple-instance learning,^33^ which has also been applied to reaction prediction problems.^34^ This is, unfortunately, not practical for our data sets due to the combinatorial explosion in the number of conformers that need to be considered. For example, the BH1 data set contains 3955 four-component reactions. Considering only ten conformers per component (certainly not enough to encompass the full conformer space for many molecules) yields 10,000 possible conformer states for each reaction and an overall data set size of more than 39 million rows. The 2D-based PhysChem descriptors capture interpretable molecular properties related to size, polarity, hydrogen bonding, and topological structure and are fast to calculate.^23^ Similarly fast are MFPs, which encode the molecular topology via circular substructures centered on each atom. The simplest descriptor is OHE, which provides a structure-agnostic baseline by assigning a unique categorical identifier to each reaction component. While lacking explicit chemical information, this representation allows the model to learn statistical patterns purely from identity. Further details of the descriptor calculations and ML model training are provided in Section S2 in the Supporting Information.

Figure 4 shows the predictive performance of the RF regression models trained with the five molecular descriptor types across the four data sets in a 5-fold cross-validation. The cross-validation was repeated five times following the procedure described in Ash et al.^35^ to assess the statistical significance between the model differences (Figure 5). The error metrics are provided in Section S3 of the Supporting Information (Table S3). While the models trained on the DFT-derived descriptors gave the smallest error for the BH1 data set, they do not outperform the simple PhysChem or MFP descriptors on the BH2 and SL1 data sets. For the SM data set, the difference is statistically significant but very small (MAE of 7.2% versus 7.4%). Even for the BH1 data set, the improvements in MAE of the DFT model relative to models trained on simpler descriptors are modest with an MAE of 3.3% for the model trained with the DFT-derived descriptors versus an MAE of 4.5% with the model trained on MFPs. These small differences highlight the extent of information that is already contained in the MFP and PhysChem descriptors. As they require only SMILES strings and no QM calculation or geometry optimization, they provide a favorable trade-off between performance and scalability. They should therefore be generally included as a baseline in ML studies on reaction yield prediction. Note that MFP and PhysChem descriptors could also easily be combined either pre- or posttraining of the ML model.

**Figure 4:**
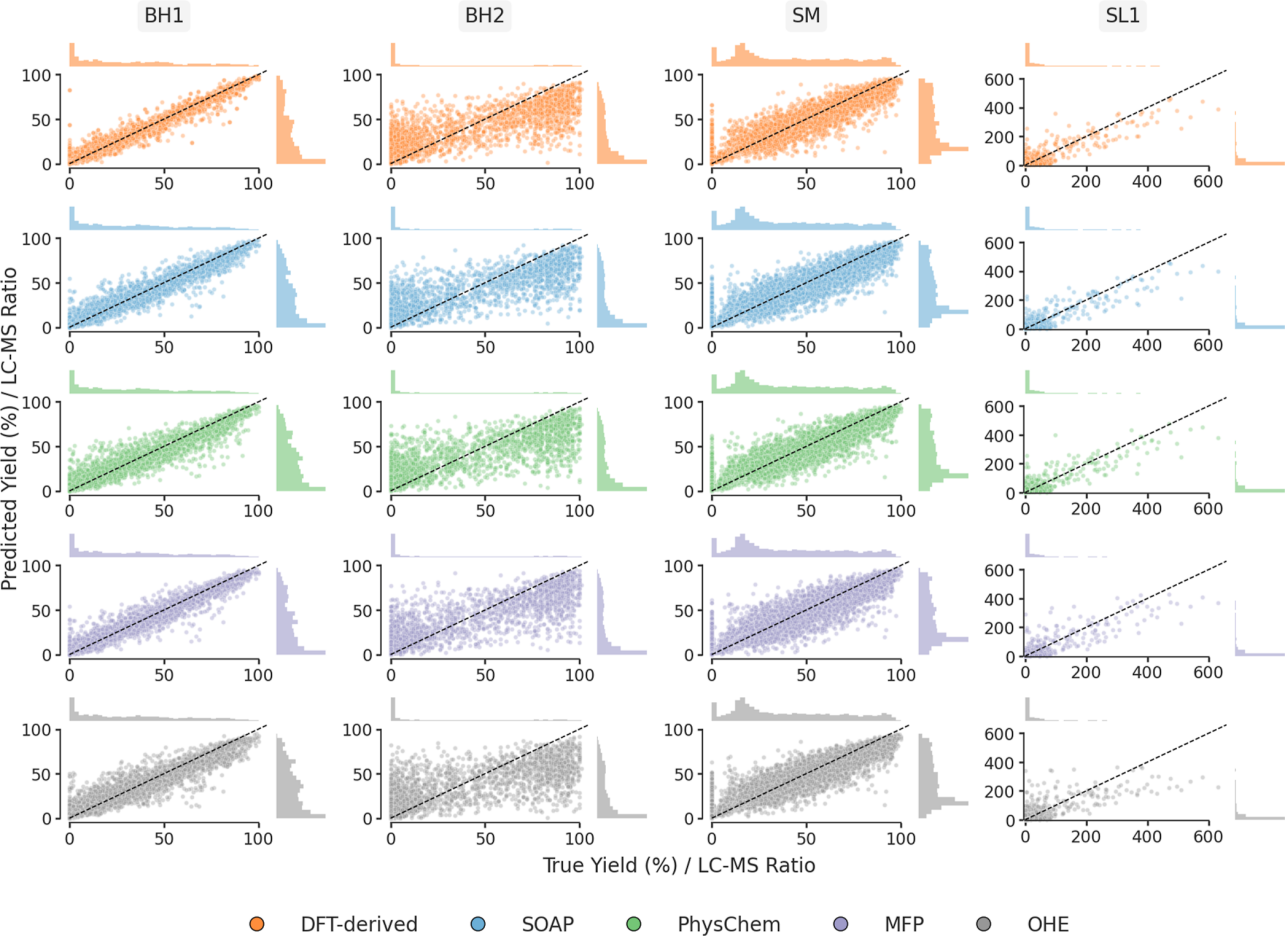
Parity plots illustrating the predictive performance of RF regression models trained on five levels of molecular descriptor complexity (DFT-derived, SOAP, PhysChem, MFP, and OHE) for four of the data sets (BH1, BH2, SM, and SL1). Predicted values are plotted against true experimental values. For BH1, BH2, and SM, the values represent reaction yields (%), whereas for SL1, the values reflect LC–MS product ratios, resulting in a different scale. Black lines indicate *y* = *x*. The histograms on top and right side show the distributions of the data points on each axis.

**Figure 5:**
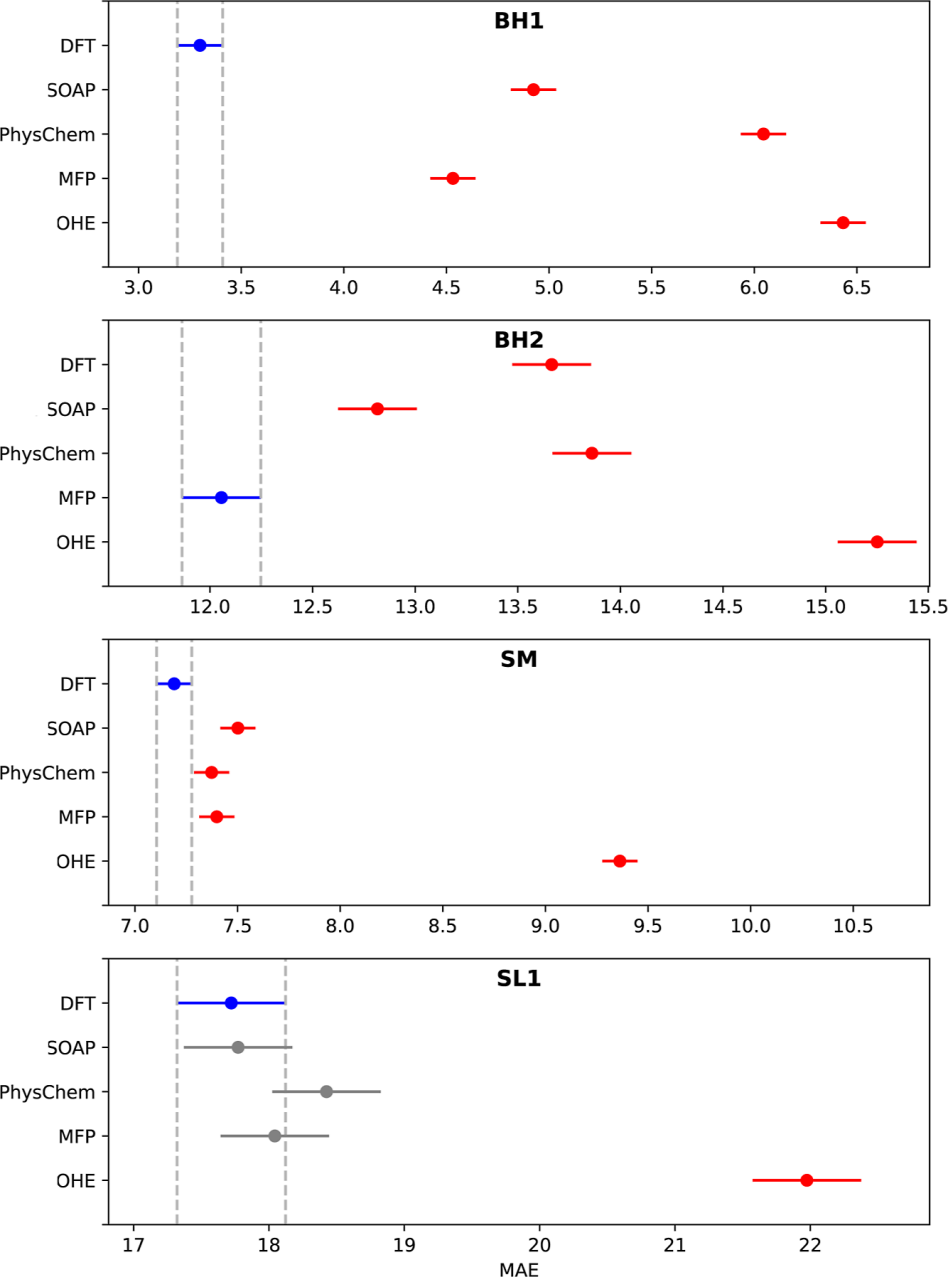
Simultaneous confidence interval plot from a 5 × 5-fold cross-validation following the procedure by Ash et al.^35^ to compare the performance of the RF models trained with the five different descriptors on the four HTE data sets. Blue indicates the best performing model with the horizontal dashed lines marking the confidence interval. Models shown in red are statistically significantly different from the best one. Gray indicates equivalent performance. For BH1, BH2, and SM, the MAE values represent reaction yields (%), whereas for SL1, the values reflect LC–MS product ratios, resulting in a different scale.

The 3D information encoded in the SOAP descriptors (which necessitates the computational cost of 3D conformer generation and optimization) does not appear to be useful for these HTE data sets. The corresponding RF models perform on par with the models trained on MFP or PhysChem descriptors. As expected, RF models trained on OHE descriptors, which contain no chemical information, give the highest errors; however, they still perform surprisingly well given the simplicity of the descriptors.

While the results above are for RF models, very similar findings were obtained with other ML algorithms (LightGBM,^30^ KNN,^25^ and ridge regression^25^), as shown in Section S4 in the Supporting Information.

### Comparison on the BH2 External Validation Set

Above we used the DFT-derived descriptors from ref 7 for the BH2 data set to enable a consistent comparison across all data sets. However, in the study of Denmark and co-workers,^8^ the authors proposed their own combination of high-dimensional QM-based and empirical descriptors to predict product yields. A full breakdown of their featurization protocol is provided in the Supporting Information of ref 8. To enable a direct comparison to the published results, we make use of their external validation set of 187 out-of-sample reactions, for which they published the yield predicted with their ML model for each data point.^8^ The 187 reactions fall into 13 groups (A–M) based on the product. As a simple model, we trained a RF regressor with MFPs using the full BH2 data set as the training set.

In terms of average MAE per group, the MFP-based model achieves comparable accuracy to the DFT-derived approach for several products (e.g., A, C, H, and M), while the performance gap widens for more challenging products such as E, J, and K, consistent with trends noted in the original study (Figure 6A). Some of the conclusions may, however, be a bit misleading when looking at the parity plots shown in Figure 6b. In general, both ML models often fail to predict high-yield reactions correctly. Exceptions are group G and, to some degree, groups J and K, for which the model trained on DFT-derived descriptors predicts high yields for at least some of the reactions. The MFP-trained model predicts yields of <50% for all reactions. This behavior may reflect a lack of generalizability in this model and/or the high prevalence of low- and zero-yield outcomes in the data set. In the case of group L, a low yield is the correct assumption, which explains the much smaller MAE of the MFP-trained model compared to the model trained on DFT-derived descriptors for this group.

**Figure 6:**
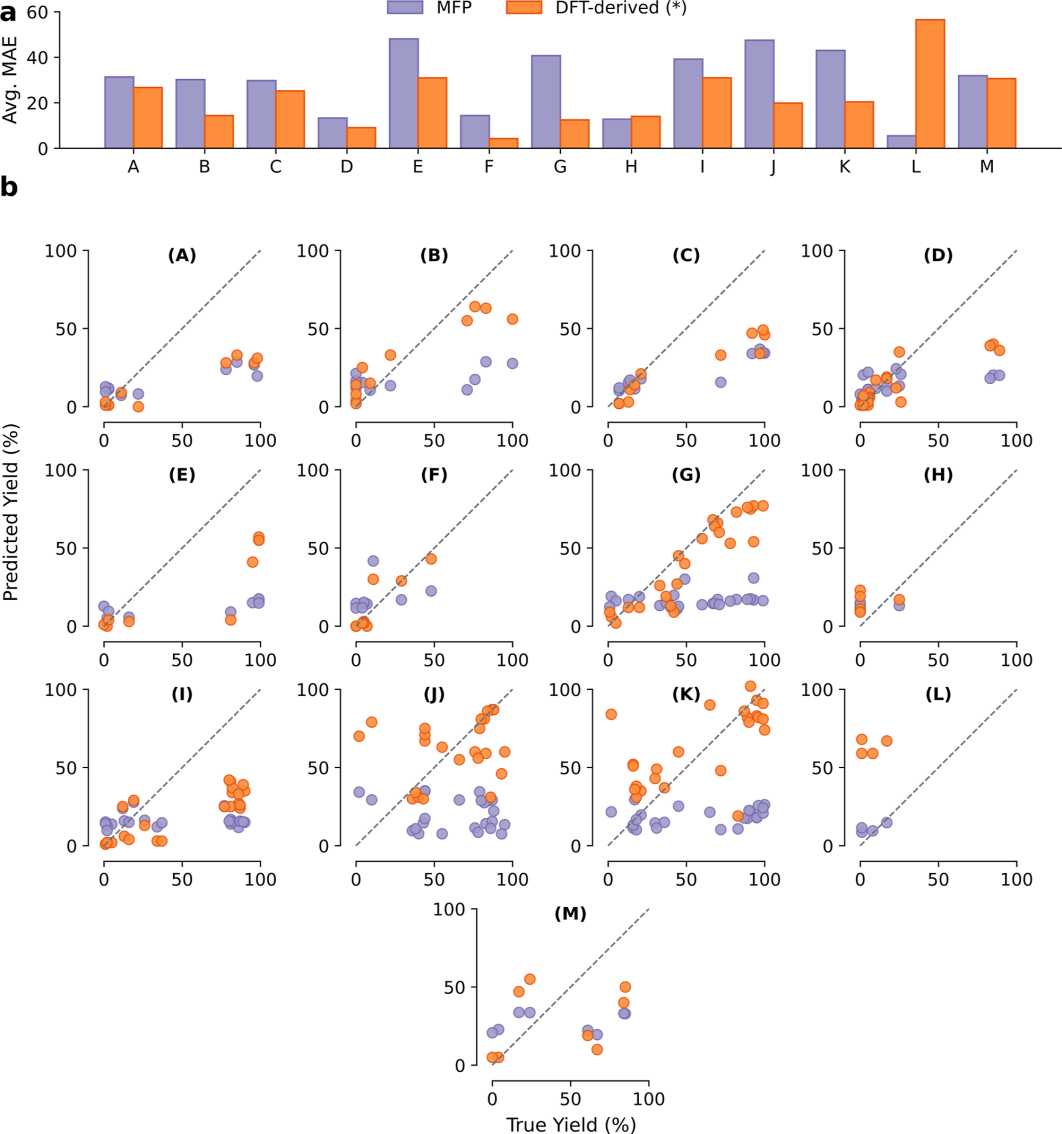
Prediction performance on the BH2 external validation set with 187 out-of-sample reactions across product groups A–M for the RF regressors trained on MFP descriptors (purple) and for the ML model from Rinehart et al.^8^ (orange), which was trained on DFT-derived descriptors (predicted yields directly taken from ref 8). (*): Note that these DFT-derived descriptors are different from those in ref 7 that were used in the rest of this study. (a) Mean absolute error (MAE) per product group. For MFP, the model was also trained on the complete training set (in contrast to the cross-validation in Figure 4). (b) Parity plots of predicted versus experimental yields per product group.

Two conclusions can be drawn from this comparison: (1) For most groups, neither model performs very well (the average MAE in the cross-validation was around 12–13%). (2) The overall performance of the MFP model is notable given the minimal computational cost. In contexts where rapid screening or scalability is a priority, MFPs offer a practical alternative to expensive QM descriptors. Especially in cases where only a “fail, no-fail” classification is required, 2D-based descriptors may be sufficient.

### How Can the Ability of a Model to Generalize to Novel Components Be Tested?

Assessing the ability of a ML model to predict the reaction yield for components not seen during training (i.e., generalization to novel chemistry) requires a different performance evaluation approach than random partitioning of data sets (as done in the cross-validation above). One possibility is to use a component-based splitting protocol as described in the work of Götz et al.^10^ Three (or more depending on the number of reaction components) “dimensionality splitting” strategies are defined with increasing generalization difficulty (Figure 7):

(i) 0D split (“in distribution”) allows all unique molecular components to appear in both the training and test sets. This corresponds to a typical random split.
(ii) 1D split (single-component holdout) holds all data points involving a given instance of a component type (e.g., aryl halides or ligands) from the training data. This simulates a scenario in which a chemist encounters a new substrate within a known reaction class and wishes to infer its reactivity from prior examples.
(iii) The 2D split (pairwise-component holdout) excludes a given pair of instances of two components (e.g., a particular aryl halide and a particular ligand) from the training set. This represents the most challenging generalization task, predicting the outcome of entirely unseen substrate pairs.

**Figure 7:**
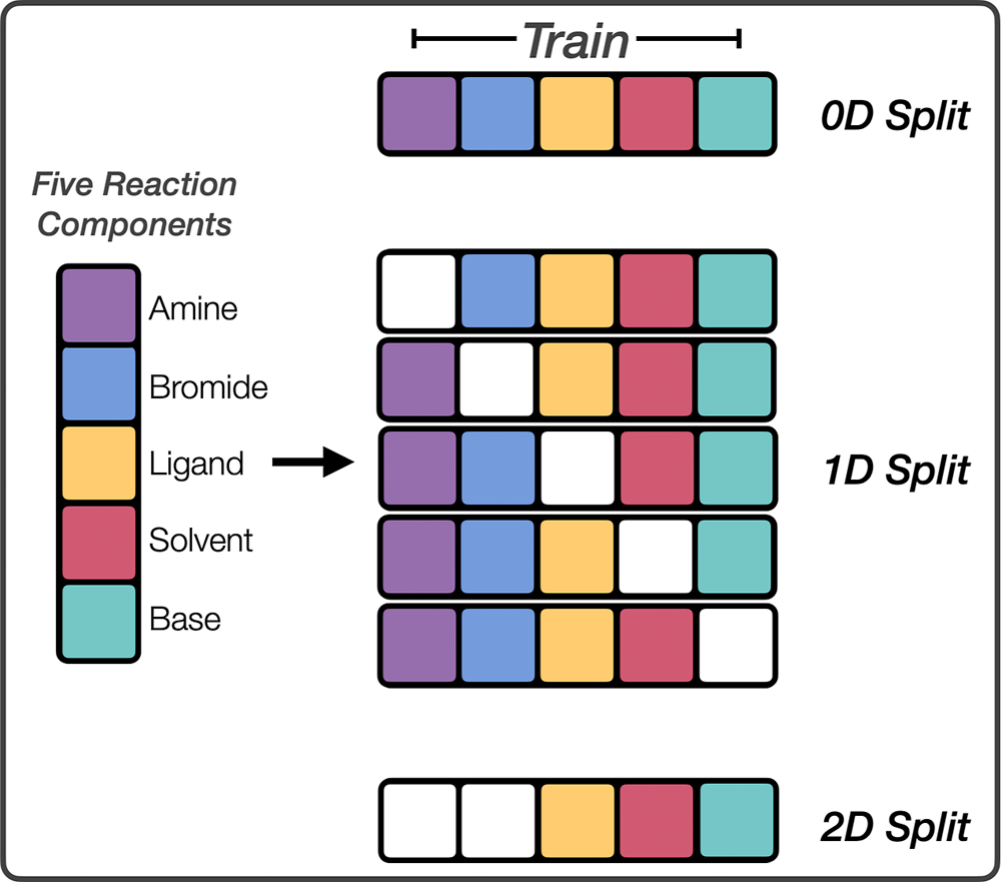
Schematic illustration of dimensionality splitting strategies. For reactions comprising five components, the 0D (in-distribution) split randomly partitions reactions such that all unique molecules of each component type are present in both the training and test sets. In the 1D (single-component holdout) split, a subset of unique molecules from one component type is withheld entirely from the training data and appears only in the test set, indicated by the absence of that component color (shown in white). In the 2D (pairwise-component holdout) split, a subset of unique molecule pairs from two component types is withheld together, such that specific combinations (e.g., amine–bromide pairs) are present exclusively in the test set.

Considering the five descriptors investigated above, we would assume that more complex (and more chemically meaningful) descriptors perform better in generalizing to new chemical matter than simpler ones. While this is certainly true for the OHE descriptor (it naturally fails for 1D and 2D splits as it contains no chemical information), models built using 2D topology-based descriptors like MFP may contain sufficient information to generalize under these conditions. For example, in the work by Götz et al.^10^ on the SL1 data set, the authors did not observe a meaningful difference in performance between MFP and QM-derived descriptors when evaluating dimensionality splits.

An important point to realize is, however, that if the combinatorial grid of possible reaction conditions is incomplete (i.e., there is not (yet) data for all possible combinations of components available), the 1D (and higher) dimensionality splitting strategy is nontrivial to perform correctly. To illustrate the issue, we consider the 1D case. To construct the test set, a subset of unique molecules for a given reaction component (e.g., a specific amine, bromide, ligand, base, or solvent) must be selected, and all reactions in which they occur are assigned to the test set. However, because the combinatorial grid is incomplete, molecules appear with highly uneven frequencies. Consequently, holding out one example of a component (e.g., a single amine) may remove only a small portion of the data set, whereas holding out another may eliminate a much larger fraction, leading to train/test splits that vary substantially in size and difficulty. Thus, calculating evaluation metrics like MAE or RMSE over different random 1D hold-out sets within the same component class average over different numbers of data points, making them difficult to meaningfully compare. In addition, the performance may vary substantially between different holdout sets depending on the chemical similarity of its members to the molecules in the training set. These effects can be directly seen in Figure 6. One solution may be to select only molecules for the hold-out set that have a high (and comparable) number of reactions in the data set, but such a procedure may introduce other biases if the subsampling of the combinatorial grid to produce the original data set was not random (e.g., a particular ligand, base, or solvent is overrepresented because it is cheap or often produces high yields, etc.). The same issues arise with higher-dimensional splits, likely even more severe.

In summary, it is important to go beyond random training/test splits when assessing model performance if the model is expected to be used on novel components. However, employing dimensionality splitting strategies in combination with an incomplete data set requires a careful analysis of the selected hold-out sets and possible biasing factors to avoid too optimistic or too pessimistic conclusions.

### Can the Imbalance in Reaction Yield Data Sets Be Addressed?

As discussed in the Introduction, the current reaction yield data sets are asymmetric, i.e., they are biased toward either high-yield reactions or low-yield reactions (Figure 2). This imbalance poses a fundamental challenge for ML both during training and assessment of the performance of a model. For classification models, class imbalance is a known issue,^36,37^ and multiple approaches to resolve it are available (e.g., undersampling, SMOTE,^38^ GHOST,^39^ etc.). The issue is less studied for regression models and has been largely ignored in ML studies of reaction-yield prediction. However, it may be especially relevant for HTE data sets where the underrepresented regime is the desired high-yield reactions.

#### Model Evaluation

Standard regression evaluation metrics such as MAE or RMSE assume symmetric error distributions and equal importance across the label space. To illustrate why this may be a problem with imbalanced data sets, let us look at product groups A and D in Figure 6b. Product group A contains a similar number of data points with high yields and low yields. While the ML models predict the ones with low yields correctly, they are very off for the data points with high yield. The MAE values shown in Figure 6a are accordingly high (∼30%). Product group D, on the other hand, is imbalanced, containing more data points with a low yield than a high yield. Again, the model performances are good for low yield and bad for high yield, but due to the imbalance, the MAE values are much smaller (around 15%).

For MAE and RMSE metrics, this issue can be resolved by using weighted variants.^40,41^ The main idea here is to assign a relevance value (or weight) to each data point that reduces the importance of data in overrepresented regions and increases the importance of data in underrepresented regions. The challenge lies in the definition of appropriate criteria for over- and underrepresentation. Preliminary investigations with the HTE data sets used here found that the procedure in ref 40 is not straightforward to apply due to the nature of the data distributions (data not shown). There are numerous other approaches discussed in ref 40, and this is an active area of research.

#### Model Training

Asymmetric data also pose an issue when training a model because bad performance in underrepresented yield regimes may not be penalized enough, resulting in poor calibration. Different approaches have been proposed to address this.^40^ One possibility from the field of reaction-yield prediction is the cost-sensitive hybrid weighting strategy of Ma et al.,^42^ which integrates focal loss with label distribution smoothing (LDS). LDS mitigates label imbalance by reweighting samples according to a smoothed estimate of the empirical yield distribution (obtained via kernel density estimation), thereby upweighting underrepresented yield regions (e.g., high yields in HTE data sets), whereas the focal loss strategy dynamically scales the loss based on the prediction difficulty. We explored the impact of applying the cost-sensitive hybrid weighting strategy to RF models of the four HTE data sets. The results demonstrate that this reweighting approach does not have a statistically significant impact on model quality, as measured by MAE, for the data sets investigated here (Section S5 of the Supporting Information). This is, of course, just one reweighting strategy, and we are evaluating it using an unweighted quality metric.

In summary, the impact of data set imbalance when training, testing, and applying ML models to predict reaction yields is currently a research problem that needs further investigation and development.

## Conclusions and Recommendations

Despite growing interest and progress in ML-based yield prediction, our findings challenge the notion that this is a “solved problem.” Even when models perform well under conventional validation, critical limitations emerge under more realistic constraints, such as distributional asymmetry, component-wise generalization, and transfer to external data sets. As our analysis shows, simple models using 2D-based descriptors (e.g., MFP and PhysChem) can rival more complex approaches in overall accuracy, in contrast to conventional expectations. The substantially higher computational costs associated with the generation of more complex descriptors need to be justified through statistically significant (but also practically relevant!) performance improvement.

We advocate for the following good practices to guide future work in this space:

1. Avoid unnecessary descriptor complexity and include simple baselines. Simpler models and descriptors can provide strong baselines and should always be included in a study for comparison. Parsimony should be prioritized, unless there is a clear, data-driven reason to invoke greater descriptor complexity.
2. Benchmark meaningfully. Evaluation protocols that reflect real-world application scenarios should be used, such as component-wise data splits and external validation data sets. High performance on narrow or biased data sets may obscure poor generalization ability. Yield prediction must be evaluated under diverse, chemically realistic conditions.
3. Acknowledge and, if possible, address data imbalance. Distributional skew in the data sets remains an unsolved challenge for both building and evaluating predictive models. While simple weighting schemes may not provide the desired fix, the problem itself is real and demands continued attention and more research. In the meantime, performance across the yield distribution can be reported rather than relying solely on overall averages, since skewed data sets can make models appear accurate while failing to identify the high-yielding reactions that are of greater practical importance.

Finally, we should always remember that the reaction yield is not a purely structural property. Without information about reaction conditions, stoichiometry, or competitive pathways, the applicability of predictive models to reactions beyond those in their training sets is questionable. Making progress here requires reaction data sets with more complete and consistently captured metadata.

Looking forward, we urge the community to resist the temptation to treat reaction-yield prediction as a settled task. As models become increasingly embedded in automated workflows and synthesis planning tools, their reliability across chemical space and across the full yield spectrum is more important than ever. A renewed focus on generalization, interpretability, pragmatic model design, and thorough performance assessment will be key to building tools that are not just accurate but trustworthy and practically useful.

## Supplementary Material



## Notes

Details on the reaction yield data sets and methods, additional results for the cross-validation, and comparison of classical ML algorithms (PDF)

## Notes

Data and Code Availability: The supporting data and source code for our study are available at the ETH Research Collection: 10.3929/ethz-c-000800856.
