# Supplementary material for: Yield Smarter, Not Harder: Good Practices for Machine Learning of Reaction Outcomes
Source: J Am Chem Soc. 2026 Jul 22;148(30):31593–603. doi: 10.1021/jacs.6c02213 (PMC13449950; doi:10.1021/jacs.6c02213)
Supplement: Supplementary file 1 [file ja6c02213_si_001.pdf]

SUPPORTING INFORMATION
Yield Smarter, Not Harder: Good Practices for Machine Learning of Reaction Outcomes

Idil Ismail,[a] Gregory A. Landrum,[a] and Sereina Riniker[a]*

[a] *Department of Chemistry and Applied Biosciences, ETH Zürich, Vladimir-Prelog-Weg 2, 8093 Zürich, Switzerland. E-mail: sriniker@ethz.ch*

Contents

S1 Reaction Yield Datasets **S2**

S2 Methods **S2**
- S2.1 HTE Datasets . . . S2
- S2.2 Descriptor Calculation . . . S3
  - S2.2.1 DFT-Derived Descriptors . . . S3
  - S2.2.2 SOAP . . . S3
  - S2.2.3 2D Physicochemical Descriptors . . . S4
  - S2.2.4 Count-Based Morgan Fingerprints . . . S4
  - S2.2.5 One Hot Encoding . . . S4
- S2.3 Training Details of RF Regression Models . . . S4
- S2.4 Training Details of Other ML Algorithms . . . S5
- S2.5 Comparing Model Performance . . . S5

S3 Additional Results for Cross-Validation of RF Regression Models **S6**

S4 Comparison of Classical ML Algorithms **S6**

S5 Reweighting Experiments for ML Model Training **S11**

S1 Reaction Yield Datasets

For each dataset, the complete list of unique SMILES strings used for each reaction component is provided as individual `csv` files. These component SMILES were extracted directly from the corresponding reaction dataset (`<Dataset>.csv`) by collecting all unique entries from each relevant column. The files are available in the ETH Research Collection: `https://doi.org/10.3929/ethz-c-000800856`.

A summary of the reaction components comprised in the four datasets is given in Table S1.

Table S1: Summary of building blocks in the four datasets.

Dataset	Components
**BH1**	15 Aryl halides; 22 Additives; 3 Bases; 4 Catalyst ligands
**BH2**	32 Amines; 37 Bromides; 20 Catalyst ligands; 3 Solvents; 3 Bases
**SM**	7 Electrophiles; 4 Nucleophiles; 11 Catalyst ligands; 7 Bases; 4 Solvents
**SL1**	40 Aldehyde-1; 5 Bifunctional reagents; 60 Aldehyde-2

S2 Methods

S2.1 HTE Datasets

We focused on four publicly available datasets spanning three reaction classes: Buchwald-Hartwig amination (datasets BH1[1] and BH2[2]), Suzuki-Miyaura coupling (dataset SM[3]), and the SLAP reaction (datasets SL1[4]).

Buchwald-Hartwig (BH) amination is a palladium-catalyzed cross-coupling of amines with aryl or heteroaryl halides to form carbon–nitrogen (C–N) bonds, widely employed for its efficiency and selectivity in pharmaceutical and agrochemical synthesis.[5] Suzuki-Miyaura (SM) coupling, also palladium-catalyzed, forms carbon–carbon (C–C) bonds via the reaction of organoboron compounds with aryl halides, and is noted for its broad functional group tolerance.[6] The SLAP reaction, as a third example, represents a mechanistically distinct, tin-free, visible-light–driven method for constructing saturated N-heterocycles. It involves the photocatalytic cyclization of bifunctional SLAP reagents, formed in situ from one aldehyde and a bifunctional core, with a second aldehyde, affording substituted, N-unprotected piperazines.

The BH1 dataset from the work of Doyle and co-workers[1] contains 3,955 reactions, all conducted in DMSO using p-toluidine as the amine coupling partner, with systematic variation across 15 aryl and heteroaryl halides, 4 Buchwald-type ligands, 3 inorganic bases, and 22 isoxazole additives. The BH2 dataset was published by the Denmark group,[2] comprising 3,359 reactions. This dataset features 121 amine–bromide reactant pairs evaluated under 24 reaction conditions, sampled from a larger design space of 180 possible combinations involving 20 palladium ligands, 3 solvents, and 3 bases. The SM dataset[3] extends the scope to C–C coupling and comprises 5,760 reactions sampled from a combinatorial space of 8,624 conditions, generated by varying 7 electrophiles, 4 nucleophiles, 11 palladium ligands, 7 bases, and 4 solvents. In this work, we removed rows from this dataset where information about either a ligand or other reagent is missing, resulting in a subset of 4,620 reactions where all components are specified and present. The SLAP dataset (SL1) published by Bode and co-workers[4] contains 1,150 reactions from the combinations of 50 aldehydes, 5 bifunctional SLAP reagents, and 60 structurally diverse aldehyde partners. There was no variation in reaction conditions.

S2.2 Descriptor Calculation

The molecular descriptors used in this study are: (i) density functional theory (DFT)-derived descriptors,[7] (ii) smooth overlap of atomic positions (SOAP)[8], (iii) 2D physicochemical (PhysChem) descriptors,[9] (iv) Morgan fingerprint count vectors (MFP)[10], and (v) one-hot encoding (OHE).[11]

S2.2.1 DFT-Derived Descriptors

For the BH1 dataset, the DFT-derived descriptors were taken directly from the original study,[1] where they used the Spartan software[12] at the B3LYP/6-31G*[13,14] level of theory. This level of theory was appropriate for the relatively small and neutral organic molecules in the BH1 dataset, which are primarily composed of first- and second-row elements (C, H, N, O, F, Cl, Br, and I). In contrast, the SM and SL1 datasets involve significantly greater chemical complexity, including organosilicon, organostannane, and organoboron reagents; charged inorganic species; and large, flexible phosphine ligands with multiple aryl or alkyl substituents. To accurately capture the electronic and steric properties of these systems, including polarization effects, heavier atoms, and charge delocalization, we used a larger and more flexible basis set level of theory. DFT calculations for the compounds in the BH2, SM, and SL1 datasets were carried out using the ORCA quantum chemistry package (version 6.1)[15], employing the B3LYP functional[13] and def2-TZVP basis set[16], with geometry optimization, vibrational frequency analysis, and CHELPG charge calculations[17] enabled.

For each molecule, we computed 33 descriptors proposed in Ref. 1 to capture key aspects of electronic structure, vibrational motion, and charge distribution. Electronic features included the energies of the highest occupied and lowest unoccupied molecular orbitals (HOMO and LUMO), from which the HOMO–LUMO gap, Mulliken electronegativity ($-\frac{1}{2}(E_{\mathrm{HOMO}}+E_{\mathrm{LUMO}})$), and chemical hardness ($\frac{1}{2}(E_{\mathrm{LUMO}}-E_{\mathrm{HOMO}})$) were derived. Vibrational descriptors comprised the frequencies and infrared intensities of the first ten normal modes obtained from harmonic frequency analysis. Charge-based descriptors included the maximum, mean, minimum, and standard deviation of atomic partial charges computed via both CHELPG and Mulliken population analyses[17,18]. A comprehensive list of all DFT-derived descriptors used in this study is provided in Table S2.

Table S2: Complete list of the DFT-derived descriptors calculated in this study following the approach in Ref. 1. Descriptors marked with an asterisk (*) denote atom-specific quantities extracted from the CHELPG charge map.

Category	Descriptors
**Frontier orbitals and reactivity**	E_{HOMO}, E_{LUMO}, electronegativity, hardness
**Dipole and electrostatics**	Dipole moment; *electrostatic charges
**Vibrational modes**	V1–V10 frequencies and intensities

S2.2.2 SOAP

For the calculation of the 3D SOAP descriptors[8] for each dataset, we used a cutoff radius of 6.0 Å, six radial basis functions, spherical harmonics up to degree 4, and a Gaussian smoothing parameter of $\sigma = 0.5$. Features were averaged across atoms and compressed using the `mu2` scheme introduced by Darby and co-workers.[19] To generate the `XYZ` coordinates, approximately 500 conformers were generated per molecule using the srETKDGv3 conformer generator[20] implemented in the RDKit (version 2025.03.6)[9],

the conformers were pruned using an RMSD threshold of 0.5Å, and the lowest-energy conformer was selected after optimization with either MMFF94[21] or UFF[22] (for molecules that do not have MMFF94 parameters).

S2.2.3 2D Physicochemical Descriptors

The 2D physicochemical (PhysChem) descriptors were calculated using RDKit (version 2025.03.6).[9] For each reaction component, we identified all unique molecules, evaluated all 217 descriptors across these molecules, retained only those descriptors that were numerically stable (no NaN values) and that had a non-zero variance, and then did a principal components analysis on the z-scaled descriptors. We kept a number of components sufficient to account for 90% of the variance as the final descriptor set for that component.

S2.2.4 Count-Based Morgan Fingerprints

MFPs were generated with a radius of 3 and a 1024-bit vector length, and were calculated using RDKit (version 2025.03.6).[9] We used count-based fingerprints to preserve information about substructure frequency, which is especially relevant for symmetric or repetitive motifs that are prevalent in the datasets used in this study.

S2.2.5 One Hot Encoding

OHE provides a structure-agnostic baseline by assigning a unique categorical identifier to each reaction component. While lacking explicit chemical information, this representation allows the model to learn statistical patterns purely from identity. Encoding is performed after the train-test split and fit on the training set. We used the scikit-learn implementation of the `OneHotEncoder`.[11]

S2.3 Training Details of RF Regression Models

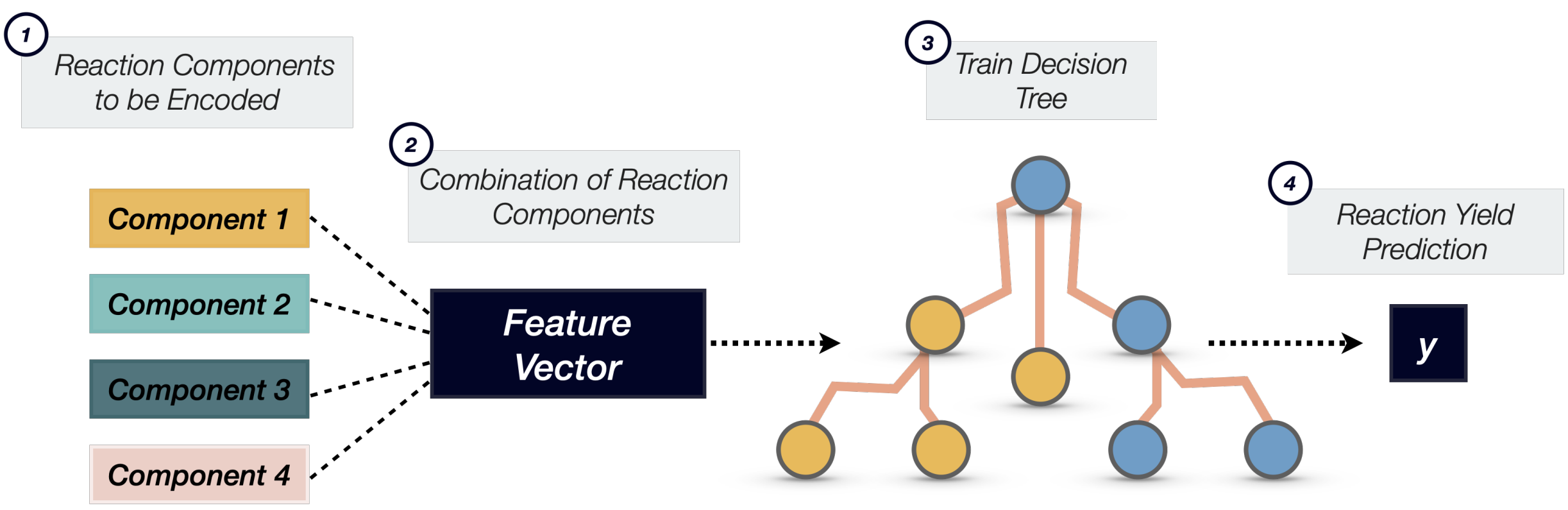

Figure S1: Workflow used in this study for reaction yield prediction using a RF regression model as ML algorithm: (1) descriptor calculation of the individual reaction components, (2) combination of the descriptors of the components into a feature vector, (3) training of the RF regression model, and (4) prediction of the reaction yield for a new instance.

For each dataset, we trained five RF regression[11] models using each of the five descriptors. As outlined in Figure S1, each model was trained on dataset-specific features corresponding to the variable reaction components (as summarized in Figure 3 in the main text). For the BH1, BH2, and SM datasets, these features included aryl halides, ligands, bases, and where applicable, solvents or nucleophiles. In the BH1 dataset, both the amine and solvent remain constant across all reactions.

For the SLAP dataset SL1, models were trained on the identities of the three coupling partners involved in the reaction. Each component was initially represented as either a SMILES string (for the MFP, PhysChem, and OHE) or an `XYZ` geometry (DFT-derived and SOAP), which was subsequently converted into a descriptor vector using one of several featurization methods described below. The resulting vectors are concatenated to form a single input per reaction, which is then used to train the RF model to predict the corresponding yield. This approach provides a consistent and interpretable baseline for assessing predictive performance across chemically diverse transformations.

All models were trained using a RF regressor implemented in scikit-learn version 1.6.1 with 500 trees.[11] Hyperparameter optimization was conducted via `GridSearchCV` with 5-fold cross-validation. Note that the `RandomForestRegressor` implementation in `scikit-learn` sets `max_features = 1` by default, which trains a bag classifier rather than a true random forest. To preserve the characteristic feature subsampling and interaction effects of classical random forests, we set `max_features` to 30% of the total number of features. The optimal parameters were: `max_depth=None`, `min_samples_leaf=1`, `max_features=0.30`, `n_estimators=500`, `min_samples_split=2`, `n_jobs=-1`.

With the optimized hyperparameters, a 5x5-fold cross-validation was carried out to assess RF regression performance with the five different descriptor types.

S2.4 Training Details of Other ML Algorithms

For comparison and additional benchmarking, we trained other ML models with the MFP: light gradient boosting machine (LightGBM),[23] k-nearest neighbors (KNN),[11] and ridge regression.[11] Similar to the RF regression models, we performed hyperparameter optimization using `GridSearchCV` with 5-fold cross-validation. The optimal parameters for LightGBM, were `n_estimators = 500`; for KNN regression, we considered up to 5 neighbors; and for ridge regression, we used the default parameters (`alpha = 1.0`, `tol = 0.0001`, `solver = "auto"`).

S2.5 Comparing Model Performance

Predictive models were compared to each other using the procedure by Ash *et al.*[24] to identify both the best model in a set and which other models show performance differences that are statistically significantly different. The Python code to do the comparisons was adapted from code provided by W. Patrick Walters[25] (IPython notebook provided at `https://github.com/PatWalters/practical_cheminformatics_posts/blob/main/adme_comparison/tukey_boxplot.ipynb`). The actual code used for this work is included in the data package for this publication in the ETH Research Collection.

S3 Additional Results for Cross-Validation of RF Regression Models

Table S3: Mean absolute error (MAE), root-mean-square-error (RMSE), correlation coefficient R^2, and Kendall τ of the predicted yield for the RF regression models trained with each descriptor across the four datasets (5x5 cross-validation). The standard deviation is given with $\pm$. For BH1, BH2, and SM, the MAE and RMSE values are in % (yields), whereas for SL1, the values reflect LC–MS product ratios.

Dataset	Descriptor	MAE	RMSE	R^2	Kendall τ
	DFT-derived	3.3 ± 0.2	5.3 ± 0.4	0.96 ± 0.01	0.88 ± 0.01
	SOAP	4.9 ± 0.3	7.3 ± 0.5	0.93 ± 0.01	0.84 ± 0.01
**BH1**	PhysChem	6.0 ± 0.3	8.8 ± 0.5	0.90 ± 0.01	0.81 ± 0.01
	MFP	4.5 ± 0.2	6.9 ± 0.5	0.94 ± 0.01	0.85 ± 0.01
	OHE	6.4 ± 0.3	9.3 ± 0.5	0.88 ± 0.01	0.80 ± 0.01
	DFT-derived	13.7 ± 0.5	19.1 ± 0.8	0.69 ± 0.02	0.67 ± 0.01
	SOAP	12.8 ± 0.5	18.2 ± 0.8	0.72 ± 0.02	0.68 ± 0.01
**BH2**	PhysChem	13.9 ± 0.5	19.5 ± 0.8	0.67 ± 0.02	0.66 ± 0.01
	MFP	12.1 ± 0.5	17.9 ± 0.8	0.73 ± 0.02	0.68 ± 0.01
	OHE	15.3 ± 0.6	21.9 ± 0.9	0.59 ± 0.03	0.61 ± 0.02
	DFT-derived	7.2 ± 0.2	10.7 ± 0.4	0.86 ± 0.01	0.77 ± 0.01
	SOAP	7.5 ± 0.2	11.1 ± 0.3	0.85 ± 0.01	0.76 ± 0.01
**SM**	PhysChem	7.4 ± 0.2	10.9 ± 0.3	0.86 ± 0.01	0.77 ± 0.01
	MFP	7.4 ± 0.2	11.0 ± 0.3	0.85 ± 0.01	0.76 ± 0.01
	OHE	9.4 ± 0.2	13.1 ± 0.3	0.78 ± 0.01	0.71 ± 0.01
	DFT-derived	17.7 ± 1.7	33.7 ± 4.8	0.78 ± 0.06	0.50 ± 0.03
	SOAP	17.8 ± 1.6	33.4 ± 4.4	0.79 ± 0.05	0.50 ± 0.04
**SL1**	PhysChem	18.4 ± 1.8	34.7 ± 5.4	0.77 ± 0.05	0.49 ± 0.04
	MFP	18.0 ± 1.8	35.1 ± 5.1	0.77 ± 0.05	0.51 ± 0.04
	OHE	22.0 ± 2.8	45.9 ± 7.6	0.61 ± 0.10	0.48 ± 0.04

S4 Comparison of Classical ML Algorithms

For MFP as descriptors, we benchmarked additional ML algorithms: LightGBM, KNN, and ridge regression (see Section S2.4 for details). The parity plots for the four datasets are shown in Figures S2-S5. Comparing the results for RF with those of the three other ML algorithms listed in Table S4, we find that across all datasets, LightGBM shows similar performance as RF regression and consistently outperforms KNN and Ridge regression. This pattern is repeated with the other descriptors, Figure S6, where the RF and LGBM models consistently perform better than the either KNN or RIDGE. The tree-based ensemble models substantially surpass the linear and distance-based baselines for all datasets and descriptors.

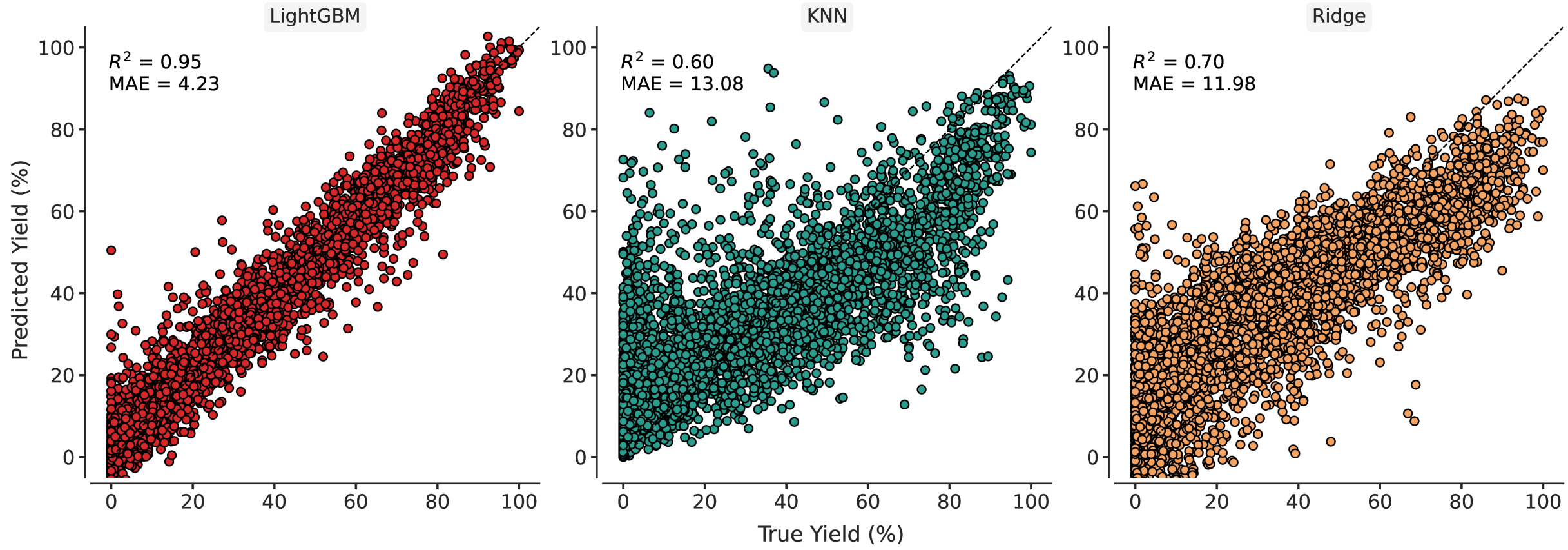

Figure S2: Parity plots of predicted versus true yields for the **BH1** dataset: (a) light gradient boosting machine (LightGBM), (b) k-nearest neighbours (KNN, $k = 5$), and (c) ridge regression trained on MFP.

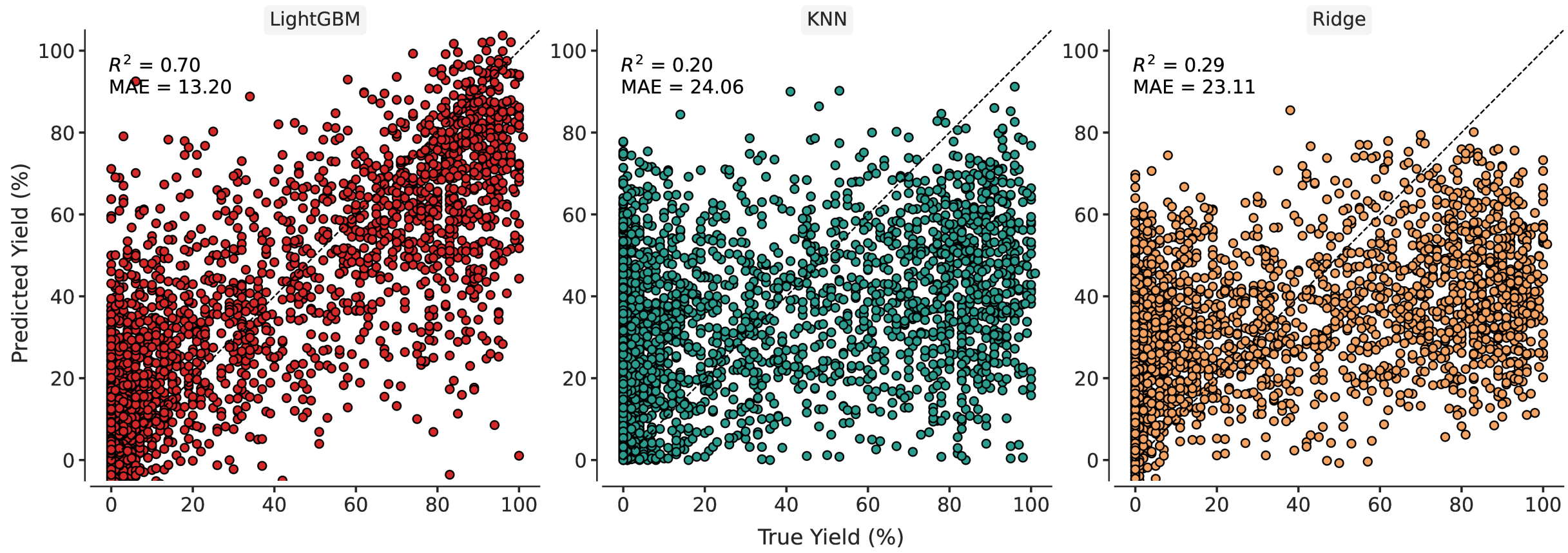

Figure S3: Parity plots of predicted versus true yields for the **BH2** dataset: (a) light gradient boosting machine (LightGBM), (b) k-nearest neighbours (KNN, $k = 5$), and (c) ridge regression trained on MFP.

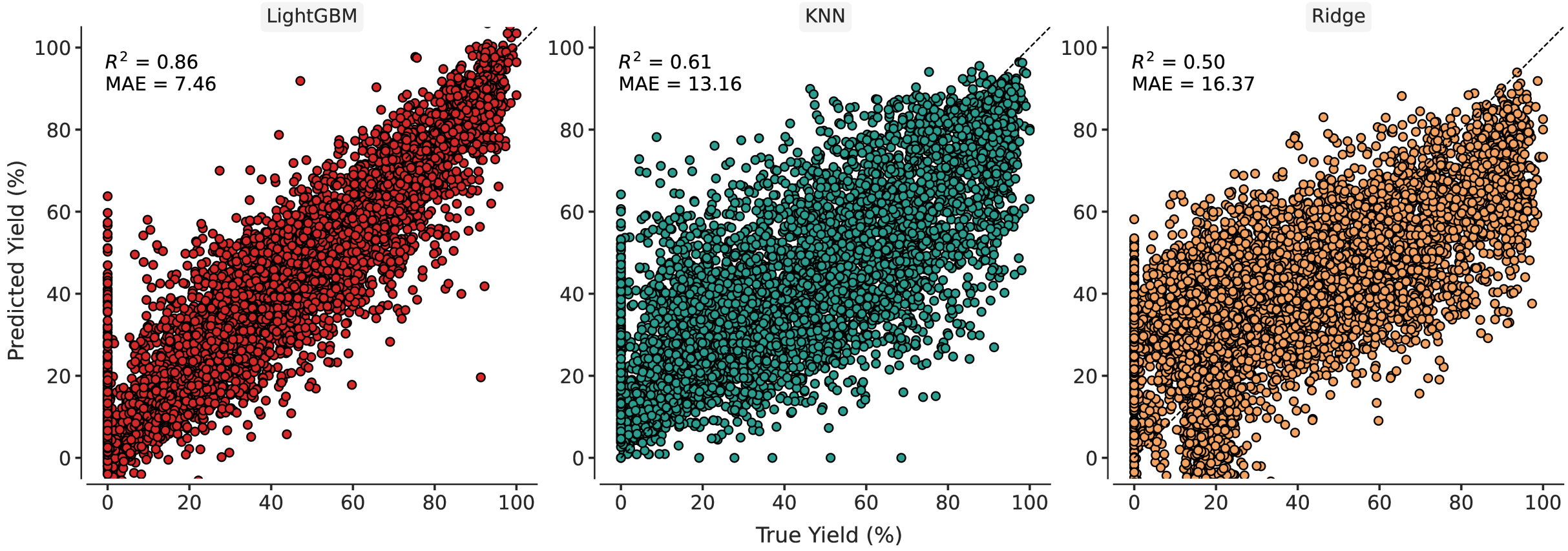

Figure S4: Parity plots of predicted versus true yields for the **SM** dataset: (a) light gradient boosting machine (LightGBM), (b) k-nearest neighbours (KNN, $k = 5$), and (c) ridge regression trained on MFP.

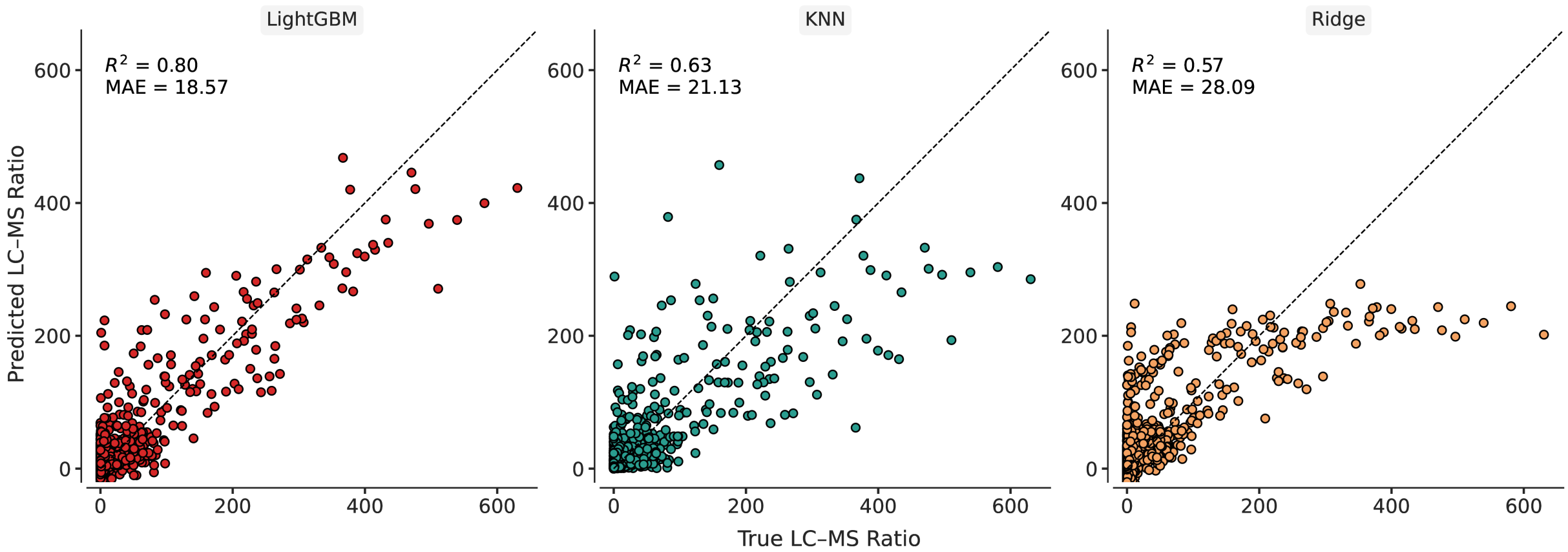

Figure S5: Parity plots of predicted versus true LC-MS Ratio for the **SL1** dataset: (a) light gradient boosting machine (LightGBM), (b) k-nearest neighbours (KNN, $k = 5$), and (c) ridge regression trained on MFP.

Table S4: 5x5-fold cross-validation performance of LightGBM, KNN, and Ridge regression models trained on MFP across four datasets (BH1, BH2, SM, and SL1). Reported metrics: mean absolute error (MAE), root-mean-square error (RMSE), coefficient of determination (R^2), and Kendall's τ. For BH1, BH2, and SM, MAE and RMSE are in % (yields); for SL1 they reflect LC–MS product ratios.

Dataset	Model	MAE	RMSE	R^2	Kendall τ
**BH1**	RF	4.5 ± 0.2	6.9 ± 0.5	0.94 ± 0.01	0.85 ± 0.01
	LightGBM	4.3 ± 0.2	6.1 ± 0.3	0.95 ± 0.01	0.86 ± 0.01
	KNN	13.0 ± 0.4	17.1 ± 0.6	0.60 ± 0.03	0.58 ± 0.02
	Ridge	12.0 ± 0.2	15.0 ± 0.3	0.70 ± 0.02	0.67 ± 0.01
**BH2**	RF	12.1 ± 0.5	17.9 ± 0.8	0.73 ± 0.02	0.68 ± 0.01
	LightGBM	13.1 ± 0.5	18.4 ± 0.8	0.71 ± 0.03	0.65 ± 0.02
	KNN	24.0 ± 0.5	30.4 ± 0.6	0.21 ± 0.03	0.37 ± 0.02
	Ridge	23.1 ± 0.6	28.8 ± 0.8	0.29 ± 0.03	0.45 ± 0.02
**SM**	RF	7.4 ± 0.2	11.0 ± 0.3	0.85 ± 0.01	0.76 ± 0.01
	LightGBM	7.4 ± 0.2	10.7 ± 0.3	0.86 ± 0.01	0.76 ± 0.01
	KNN	13.4 ± 0.4	17.8 ± 0.5	0.62 ± 0.02	0.57 ± 0.01
	Ridge	16.6 ± 0.3	20.0 ± 0.4	0.51 ± 0.02	0.52 ± 0.01
**SL1**	RF	18.0 ± 1.8	35.1 ± 5.1	0.77 ± 0.05	0.51 ± 0.04
	LightGBM	18.6 ± 1.8	32.9 ± 4.0	0.79 ± 0.06	0.47 ± 0.03
	KNN	20.7 ± 2.9	42.7 ± 7.6	0.66 ± 0.09	0.47 ± 0.03
	Ridge	28.3 ± 2.2	48.5 ± 5.5	0.56 ± 0.08	0.48 ± 0.03

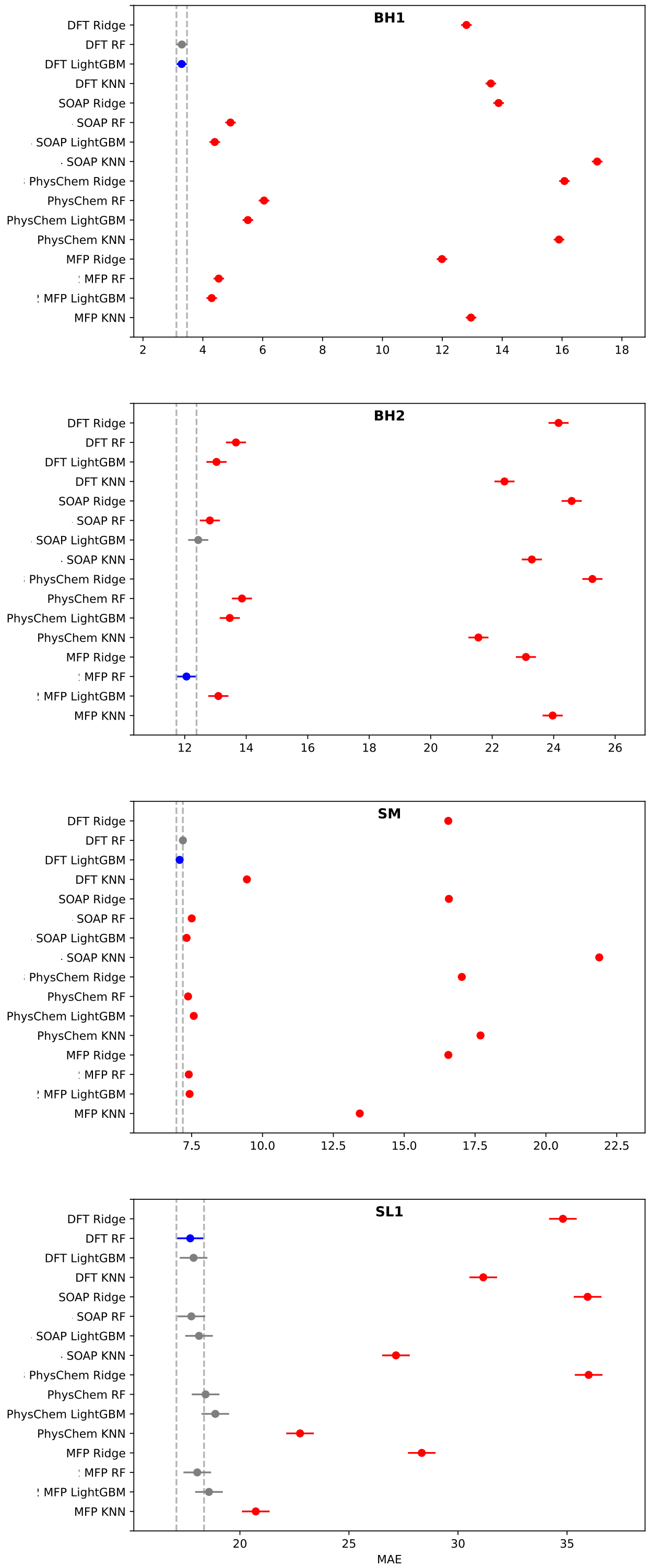

Figure S6: Simultaneous confidence interval plot from a 5x5-fold cross-validation following the procedure by Ash *et al.*[24] to compare the performance of the RF, LightGBM, KNN, and Ridge models trained with the five different descriptors on the the four HTE datasets. Blue indicates the best performing model with the horizontal dashed lines marking the confidence interval. Models shown in red are statistically significantly different from the best one. Gray indicates same performance. For BH1, BH2, and SM, the MAE values represent reaction yields (%), whereas for SL1, the values reflect LC–MS product ratios, resulting in a different scale.

S5 Reweighting Experiments for ML Model Training

We explored the impact of reweighting on model performance by applying the cost-sensitive hybrid weighting strategy of Ma *et al.*[26] to reweight the training data for RF regression models. Results for the five different descriptors and four HTE datasets are shown in Figure S7.

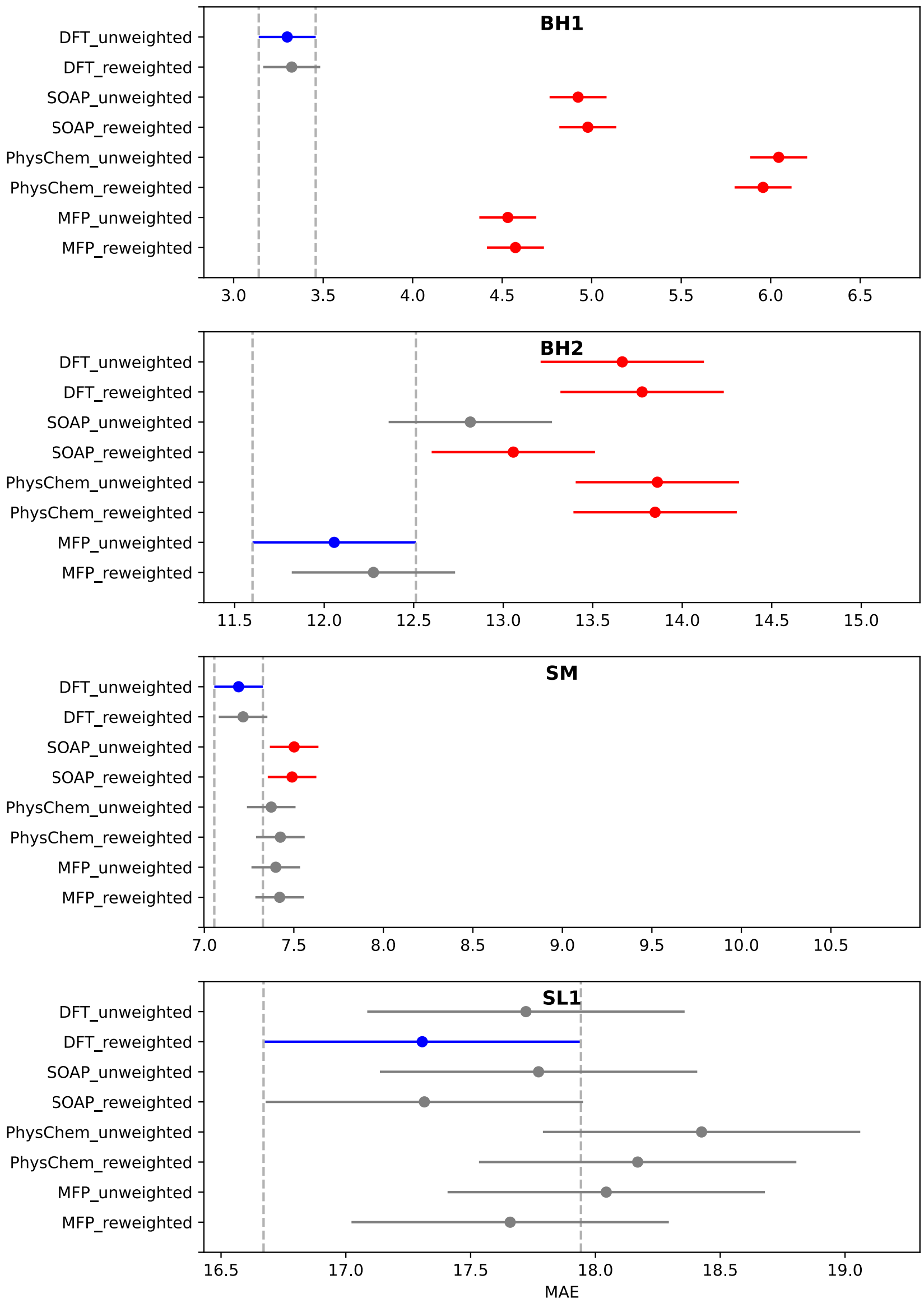

Figure S7: Simultaneous confidence interval plot from a 5x5-fold cross-validation following the procedure by Ash *et al.*[24] to compare the performance of the unweighted and reweighted training of RF models with the five different descriptors on the the four HTE datasets. Blue indicates the best performing model with the horizontal dashed lines marking the confidence interval. Models shown in red are statistically significantly different from the best one. Gray indicates same performance. For BH1, BH2, and SM, the MAE values represent reaction yields (%), whereas for SL1, the values reflect LC–MS product ratios, resulting in a different scale.

References

[1] Ahneman, D. T.; Estrada, J. G.; Lin, S.; Dreher, S. D.; Doyle, A. G. Predicting Reaction Performance in C–N Cross-Coupling Using Machine Learning. *Science* **2018**, *360*, 186–190.

[2] Rinehart, N. I.; Saunthwal, R. K.; Wellauer, J.; Zahrt, A. F.; Schlemper, L.; Shved, A. S.; Bigler, R.; Fantasia, S.; Denmark, S. E. A Machine-Learning Tool to Predict Substrate-Adaptive Conditions for Pd-Catalyzed C–N Couplings. *Science* **2023**, *381*, 965–972.

[3] Perera, D.; Tucker, J. W.; Brahmbhatt, S.; Helal, C. J.; Chong, A.; Farrell, W.; Richardson, P.; Sach, N. W. A Platform for Automated Nanomole-Scale Reaction Screening and Micromole-Scale Synthesis in Flow. *Science* **2018**, *359*, 429–434.

[4] Götz, J.; Jackl, M. K.; Jindakun, C.; Marziale, A. N.; Andr'e, J.; Gosling, D. J.; Springer, C.; Palmieri, M.; Reck, M.; Luneau, A.; Brocklehurst, C. E.; Bode, J. W. High-Throughput Synthesis Provides Data for Predicting Molecular Properties and Reaction Success. *Sci. Adv.* **2023**, *9*, eadj2314.

[5] Guram, A. S.; Rennels, R. A.; Buchwald, S. L. A Simple Catalytic Method for the Conversion of Aryl Bromides to Arylamines. *Angew. Chem. Int. Ed.* **1995**, *34*, 1348–1350.

[6] Beckers, I.; Bugaev, A.; Vos, D. D. Dual Ligand Approach Increases Functional Group Tolerance in the Pd-Catalysed C–H Arylation of N-Heterocyclic Pharmaceuticals. *Chem. Sci.* **2023**, *14*, 1176–1183.

[7] Neese, F. The ORCA Program System. *WIRES Comput. Molec. Sci.* **2012**, *2*, 73–78.

[8] Bartók, A. P.; Kondor, R.; Csányi, G. On Representing Chemical Environments. *Phys. Rev. B* **2013**, *87*, 184115.

[9] Landrum, G. et al. rdkit/rdkit: 2025_03_5 (Q1 2025) Release. 2025; `https://zenodo.org/records/16439048`.

[10] Rogers, D.; Hahn, M. Extended-Connectivity Fingerprints. *J. Chem. Inf. Model.* **2010**, *50*, 742–754.

[11] Pedregosa, F. et al. Scikit-learn: Machine Learning in Python. *J. Mach. Learn. Res.* **2011**, *12*, 2825–2830.

[12] Wavefunction, I. SPARTAN'14. 2024; `https://wavefun.com/spartan`.

[13] Becke, A. D. Density-Functional Thermochemistry. III. The Role of Exact Exchange. *J. Chem. Phys.* **1993**, *98*, 5648–5652.

[14] Hehre, W. J.; Ditchfield, R.; Pople, J. A. Self—Consistent Molecular Orbital Methods. XII. Further Extensions of Gaussian—Type Basis Sets for Use in Molecular Orbital Studies of Organic Molecules. *J. Chem. Phys.* **1972**, *56*, 2257–2261.

[15] Neese, F. Software Update: The ORCA Program System – Version 6.0. *Wiley Interdiscip. Rev. Comput. Mol. Sci.* **2025**, *15*, e70019.

[16] Weigend, F.; Ahlrichs, R. Balanced Basis Sets of Split Valence, Triple Zeta Valence and Quadruple Zeta Valence Quality for H to Rn: Design and Assessment of Accuracy. *Phys. Chem. Chem. Phys.* **2005**, *7*, 3297–3305.

[17] Breneman, C. M.; Wiberg, K. B. Determining Atom-Centered Monopoles from Molecular Electrostatic Potentials. The Need for High Sampling Density in Formamide Conformational Analysis. *J. Comput. Chem.* **1990**, *11*, 361–373.

[18] Mulliken, R. S. Electronic Population Analysis on LCAO–MO Molecular Wave Functions. I. *J. Chem. Phys.* **1955**, *23*, 1833–1840.

[19] Darby, J. P.; Kermode, J. R.; Csányi, G. Compressing Local Atomic Neighbourhood Descriptors. *npj Comput. Mater.* **2022**, *8*, 166.

[20] Wang, S.; Witek, J.; Landrum, G. A.; Riniker, S. Improving Conformer Generation for Small Rings and Macrocycles Based on Distance Geometry and Experimental Torsional-Angle Preferences. *J. Chem. Inf. Model.* **2020**, *60*, 2044–2058.

[21] Halgren, T. A. Merck Molecular Force Field. I. Basis, Form, Scope, Parametrisation, and Performance of MMFF94. *J. Comput. Chem.* **1996**, *17*, 490–519.

[22] Rappe, A. K.; Casewit, C. J.; Colwell, K. S.; Goddard, W. A. I.; Skiff, W. M. UFF, a Full Periodic Table Force Field for Molecular Mechanics and Molecular Dynamics Simulations. *J. Am. Chem. Soc.* **1992**, *114*, 10024–10035.

[23] Shi, Y.; Ke, G.; Soukhavong, D.; Lamb, J.; Meng, Q.; Finley, T.; Wang, T.; Chen, W.; Ma, W.; Ye, Q.; Liu, T.-Y.; Titov, N.; Cortes, D. lightgbm: Light Gradient Boosting Machine. 2025; R package version 4.6.0.99.

[24] Ash, J. R.; Wognum, C.; Rodríguez-Pérez, R.; Aldeghi, M.; Cheng, A. C.; Clevert, D.-A.; Engkvist, O.; Fang, C.; Price, D. J.; Hughes-Oliver, J. M.; Walters, W. P. Practically Significant Method Comparison Protocols for Machine Learning in Small Molecule Drug Discovery. *J. Chem. Inf. Model.* **2025**, *65*, 9398–9411.

[25] Walters, W. P. Even More Thoughts on ML Method Comparisons. 2025; `https://practicalcheminformatics.blogspot.com/2025/03/even-more-thoughts-on-ml-method.html`, [Online; accessed 24. May 2026].

[26] Ma, Y.; Huang, X.; Nan, B.; Moniz, N.; Zhang, X.; Wiest, O.; Chawla, N. V. Are we Making Much Progress? Revisiting Chemical Reaction Yield Prediction from an Imbalanced Regression Perspective. Companion Proceedings of the ACM Web Conference 2024. New York, NY, USA, 2024; pp 790–793.
